# Effects of Atrazine on Reproductive Health of Nondiabetic and Diabetic Male Rats

**DOI:** 10.1155/2014/676013

**Published:** 2014-10-28

**Authors:** Dinesh Babu Jestadi, Alugoju Phaniendra, Undru Babji, Bhavatharini Shanmuganathan, Latha Periyasamy

**Affiliations:** Department of Biochemistry and Molecular Biology, School of Life Sciences, Pondicherry University, Puducherry 605 014, India

## Abstract

The aim of the present study was to investigate the effects of low dose of atrazine on reproductive system of male Wistar rats. 16 rats were divided into four groups of four animals each. Group I (nondiabetic) and group III (diabetic) animals served as controls that received safflower oil (300 *μ*L/kg bw/day), respectively. Group II (nondiabetic) and group IV (diabetic) animals received atrazine (300 *μ*g/kg bw/day). Nonsignificant decrease in the activities of antioxidant and steroidogenic enzymes and sperm parameters suggests that atrazine did not produce any effect on reproductive system of rats. Histological findings also revealed that atrazine at a dose of 300 *μ*g/kg bw did not produce any testicular toxic effects in nondiabetic and diabetic atrazine treated rats. Low dose of atrazine did not show reproductive toxicity in rats. To know the effects of atrazine in diabetic rats further studies have to be carried out with increased concentration of atrazine.

## 1. Introduction

Increased male reproductive abnormalities are due to increased exposure to environmental contaminants such as organochlorine pesticides, poly chlorinated biphenyls, dioxins, phytoestrogens, and other xenoestrogens that enter the human system through food, drinking water, air, and skin contact [1]. Pesticides have been widely used all over the world because they enable the development of agricultural and farming production by controlling a wide range of pests and diseases. However, it is well known that the application of these substances affects human health as well as the environment [2, 3]. Atrazine (2-chloro-4-ethylamino-6-isopropylamine-1,2,5-triazine: ATZ) is used to control broad-leaf weeds and grasses, for example, corn, sorghum, sugarcane, pineapple, turf, and orchards. ATZ has attracted great attention due to its widespread use and ubiquitous contamination in ground and surface waters, its pattern of use, high persistency, and its potential biological impact in the environment [4]. Atrazine decreases the sperm motility, viability, and count by inducing oxidative stress through the depletion of the antioxidant activities [5, 6]. Atrazine decreases the secretion of follicular stimulating hormone (FSH), luteinizing hormone (LH), and testosterone concentration by decreasing the weight of pituitary gland and gonadotropin-releasing hormone (GnRH) secretion from hypothalamus [7].

Diabetes mellitus (DM) is a chronic hormonal and metabolic disorder. Globally 366 million people had DM in 2011 and it is expected to rise up to 552 million in 2030. Out of the global diabetic population, 80–90% diabetic patients are suffering with type 2 diabetes mellitus (T2DM) which caused 4.6 million deaths in 2011 and is expected to reach 439 million in 2030 [8, 9]. Etiology of T2DM is very complex and influenced by different factors such as obesity, lack of physical exercise, cigarette smoking, sedentary lifestyle, alcohol consumption, and environmental toxins [8]. In India 50.8 million people have diabetes and it is expected to reach 87 million by the year of 2030 [10]. DM increases oxidative stress by depleting antioxidant activities [11, 12]. DM suppresses spermatogenesis by reducing hormones such as FSH, LH, and testosterone and also increases germ cell apoptosis [13].

The aim of the present study was to investigate the possible effects of the low dose of atrazine on reproductive function of nondiabetic and diabetic rats.

## 2. Materials and Methods

### 2.1. Chemicals

Atrazine (technical grade 98.8%) and streptozotocin were purchased from Sigma Aldrich (St. Louis, MO, USA). All the other chemicals used were of analytical grade.

### 2.2. Animals

16 Adult male Wistar rats (120–180 g) were procured and housed in plastic suspended cages and fed with normal pelletized chow and water (*ad libitum*) under standard temperature (24 ± 3°C) and photoperiod (12 hours light : 12 hours dark). The experimental animals were handled as per the guidelines of Committee for the Purpose of Control and Supervision of Experiments on Animals (CPCSEA) which were approved by the Institutional Animal Ethics Committee (IAEC approval number: PU/SLS/IAEC/2014/11, dated 20.02.2014) of Pondicherry University, Puducherry, India.

### 2.3. Induction of Diabetes

High fat diet (HFD: 58% fat, 25% protein, and 17% carbohydrate) was freshly prepared daily in sterile condition with composition of 365 g normal pelletized rat chow, 310 g lard oil, 250 g casein, 10 g cholesterol, 60 g vitamin and mineral mix, 3 g methionine, 1 g yeast, and 1 g sodium chloride per kilogram [14]. Rats were fed with HFD for three weeks. On 21st or 22nd day streptozotocin dissolved in citric acid buffer (pH 4.0) at a dosage of 35 mg/kg body weight bw/rat was administered intraperitoneally to induce diabetes. After 72 hours of STZ administration animals showing high blood glucose levels (>140 mg/dL) were considered as diabetic [15].

### 2.4. Experimental Plan

After the induction of diabetes, based on the body weight and blood glucose, the animals were divided into four groups consisting of four rats in each group. The experimental period was for 15 days.

*Group I*. Nondiabetic control rats received 300 *μ*L of safflower oil/kg bw/day.

*Group II*. Nondiabetic rats treated with 300 *μ*g of atrazine/kg bw/day suspended in safflower oil.

*Group III*. Diabetic control rats received 300 *μ*L of safflower oil/kg bw/day.

*Group IV*. Diabetic rats treated with 300 *μ*g of atrazine/kg bw/day suspended in safflower oil.

### 2.5. Evaluation of Sperm Parameters

Epididymal sperms were collected by chopping the epididymis in 5 mL of Ham's F-12 medium. The epididymal sperm viability and count was assessed according to WHO laboratory manual [16]. The sperm motility was analyzed by the method of Linder et al. [17] and Cooke et al. [18].

### 2.6. Evaluation of Blood Glucose Level and Antioxidant Activities

Blood glucose levels were determined by the kit method from Agappe Diagnostics Ltd, Kerala, India. Values were expressed as mg/dL. At the end of the treatment period, the rats were fasted overnight (12 h) and euthanized by cervical dislocation. Body weights were recorded prior to euthanization. The testes were dissected out, washed in ice-cold 1.15% KCl solution, and pat-dried and wet weight was noted. Testis tissue homogenate (10% w/v) was prepared in phosphate buffer saline (PBS-pH 7.4) and centrifuged at 10000 g at 4°C for 15 min. The supernatant was collected for assessing the activities of the antioxidant enzymes such as superoxide dismutase (SOD) activity by Marklund and Marklund method [19], Catalase (CAT) by Clairborne method [20], Glutathione peroxidase (GPx) by Rotruck et al. method [21], reduced glutathione (GSH) by Ellman method [22], and glutathione-S-transferase (GST) by Habig et al. method [23]; H_2_O_2_ generation was assayed by the method of Pick and Keisari [24] and the level of lipid peroxidation (LPO) product thiobarbituric acid (TBARS) by the method of Ohkawa et al. [25].

### 2.7. Evaluation of Steroidogenic Enzyme Activities

The activities of 3*β*-hydroxy steroid dehydrogenase (3*β*-HSD) and 17*β*-hydroxy steroid dehydrogenase (17*β*-HSD) were measured by the method of Bergmeyer, [26] and the enzyme activities were expressed as nmol of NADPH converted into NADH per minute per milligram of protein.

### 2.8. Histological Examination

Testis were fixed in 10% neutral buffered formalin (pH 6.8) and processed for histological examination by using routine paraffin-wax embedding method. Sections of 5 *μ*m were stained with hematoxylin and eosin.

### 2.9. Statistical Analysis

The results were expressed as mean ± SD for four animals each group. Statistical analyses were performed by one-way analysis of variance (ANOVA) followed by Tukey's post testing using SPSS (SPSS 16.0, SPSS Inc. UK). *P* ≤ 0.05 was considered as statistically significant.

## 3. Results

### 3.1. Effects of Atrazine on Body Weight and Testis Weight

The changes observed in the body weight and testis weight in groups II and IV were not significant when compared to group I and group III (Table 1), respectively.

### 3.2. Effects of Atrazine on Sperm Parameters

The changes observed in the sperm motility, viability, and count in groups II and IV were not significant when compared to group I and group III (Table 2), respectively.

### 3.3. Effects of Atrazine on Blood Glucose Level and Antioxidants in Testis of Normal and Diabetic Rats

Significant changes were observed in blood glucose levels in diabetic control and diabetic atrazine rats when compared to normal rats. However, no significant change in the blood glucose levels was observed between diabetic control and diabetic atrazine treated rats (Table 3). The changes observed in CAT, SOD, GPx, and GST activities in groups II and IV were not significant when compared to group I and group III (Table 3), respectively. The changes in the level of glutathione content in groups II and IV were not significant when compared with group I and group III, respectively.

### 3.4. Effect of Atrazine on Lipid Peroxidation

**Figure 1:**
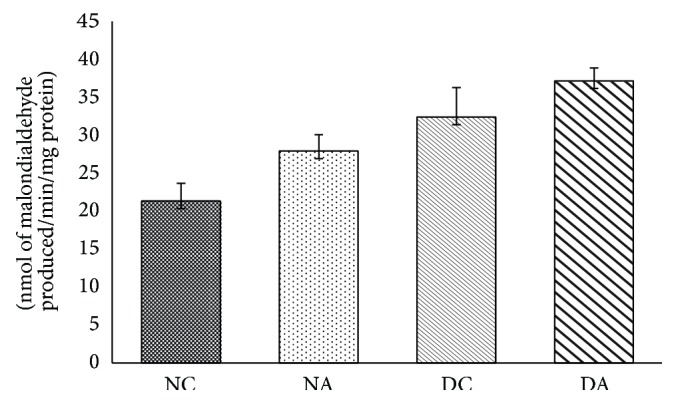
Effects of atrazine on lipid peroxidation. The data are represented as mean ± SD (*n* = 4) and evaluated by one-way analysis of variance (ANOVA) confirmed that the groups are not significantly differed (*P* > 0.05). NC = nondiabetic control rats, NA = nondiabetic atrazine treated rats, DC = diabetic control rats, and DA = diabetic atrazine treated rats.

The changes observed in groups II and IV were not significant when compared to group I and group III (Figure 1), respectively.

### 3.5. Effect of Atrazine on H_2_O_2_ Generation

**Figure 2:**
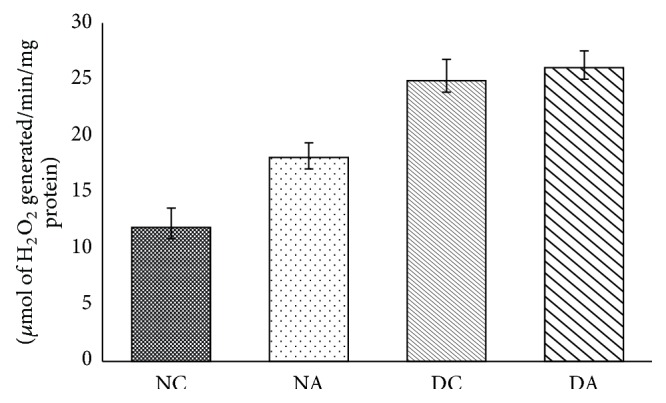
Effects of atrazine on H_2_O_2_ generation. The data are represented as mean ± SD (*n* = 4) and evaluated by one-way analysis of variance (ANOVA) confirmed that the groups are not significantly differed (*P* > 0.05). NC = nondiabetic control rats, NA = nondiabetic atrazine treated rats, DC = diabetic control rats, and DA = diabetic atrazine treated rats.

H_2_O_2_ generation in groups II and IV was not significant (Figure 2) in comparison with group I and group III, respectively.

### 3.6. Effects of Atrazine on 3*β*-HSD

**Figure 3:**
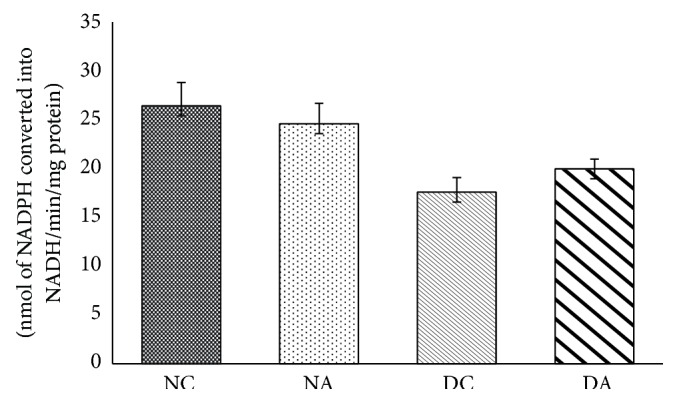
Effects of atrazine on 3*β*-HSD. The data are represented as mean ± SD (*n* = 4) and evaluated by one-way analysis of variance (ANOVA) confirmed that the groups are not significantly differed (*P* > 0.05). NC = nondiabetic control rats, NA = nondiabetic atrazine treated rats, DC = diabetic control rats, and DA = diabetic atrazine treated rats.

The changes observed in 3*β*-HSD activity were not significant in group II and group IV when compared to group I and group III (Figure 3), respectively.

### 3.7. Effects of Atrazine on 17*β*-HSD

**Figure 4:**
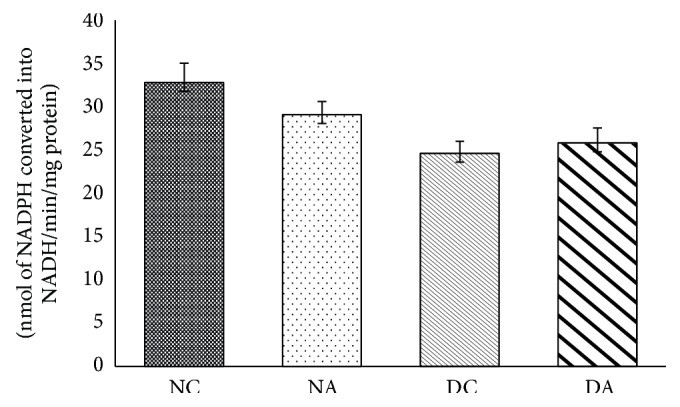
Effects of atrazine on 17*β*-HSD. The data are represented as mean ± SD (*n* = 4) and evaluated by one-way analysis of variance (ANOVA) confirmed that the groups are not significantly differed (*P* > 0.05). NC = nondiabetic control rats, NA = nondiabetic atrazine treated rats, DC = diabetic control rats, and DA = diabetic atrazine treated rats.

The changes observed in 17*β*-HSD activity were not significant in group II and group IV when compared to group I and group III (Figure 4), respectively.

### 3.8. Histological Findings

**Figure 5:**
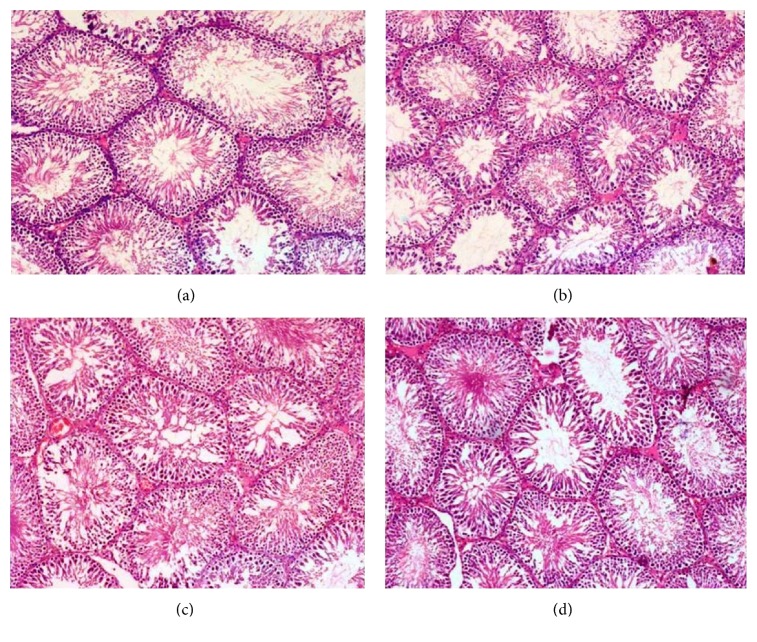
Light microscopic images of the section of testis. (a), (b), (c), and (d) are hematoxylin and eosin (H&E 40x) stained section of testis of nondiabetic control (NC), nondiabetic atrazine treated rats (NA), diabetic control rats (DC), and diabetic atrazine treated rats (DA), respectively. (a) and (b) show normal spermatogonia, Sertoli cells, and Leydig cells with accumulation of spermatozoa within the seminiferous tubules. (c) and (d) show reduced numbers of spermatogonia, Sertoli cells, and Leydig cells with drastic reduction of spermatozoa within the seminiferous tubules.

Group I, normal control rats (Figure 5(a)), shows normal spermatogonia, Sertoli cells, and Leydig cells with accumulation of spermatozoa within the seminiferous tubules. Group II shows normal atrazine treated rats (Figure 5(b)) which depicts normal spermatogonia, Sertoli cells, and Leydig cells with accumulation of spermatozoa within the seminiferous tubules. Group III, diabetic control rats (Figure 5(c)), illustrates reduced number of spermatogonia, Sertoli cells, and Leydig cells with drastic reduction of spermatozoa within the seminiferous tubules. Group IV, diabetic atrazine treated rats, shows (Figure 5(d)) reduced number of spermatogonia, Sertoli cells, and Leydig cells with drastic reduction of spermatozoa within the seminiferous tubules.

## 4. Discussion

No mortality and behavioral changes were observed in the experimental animals throughout the experimental period. Gain in the body weights was observed and it correlates with the previous reports [27–29]. The increased testes weight may be due to superficial changes because the absolute weight did not significantly differ from the control group. Increase in testicular weight was similar to the previous reports on toxicants that affect male reproductive health [30, 31].

Mammalian testis cell membranes are rich in poly unsaturated fatty acids (PUFA) and are sensitive to oxygen and nitrogen derived free radicals. Increased generation of reactive oxygen species (ROS) subjects the cell to oxidative stress and damages the cellular DNA, proteins, and lipids (lipid peroxidation) which leads to cell death [32, 33]. Increased lipid peroxidation indicates increased generation of oxygen free radicals and it is associated with decreased sperm motility, viability, and count [34, 35]. The increased level of lipid peroxidation and H_2_O_2_ reveals that atrazine affects spermatogenesis [36].

Significant changes were observed in blood glucose levels in diabetic control and diabetic atrazine rats when compared to normal rats. However, no significant change in the blood glucose levels was observed between diabetic control and diabetic atrazine treated rats. The study conducted by Lim et al. [37] with chronic administration of low dose of atrazine 300 *μ*g/kg bw/day decreased basal metabolic rate and insulin resistance. Though the dosage was same, in the present study, no significant changes in blood glucose levels were observed in diabetic atrazine rats which may be due to short treatment period (15 days). ATZ has been shown to induce oxidative stress by producing ROS [5, 38]. Exposure of experimental animals to pesticides is known to induce lipid peroxidation in various tissues, which is responsible for the adverse biological effects [39–41]. SOD converts superoxide radical into H_2_O_2_, which gets degraded by catalase and glutathione peroxidase/reductase system [42]. Reduced activities of SOD, CAT, and GPx in nondiabetic atrazine treated animals show an inability to eliminate superoxide radicals in testis. GSH plays an important role in protecting tissue from various xenobiotics induced injury [43]. But decreased levels of GSH were observed upon exposure to different pesticides [44, 45]. An increased activity of GST suggests an increased production of reduced glutathione metabolites on exposure to pesticides [40, 45]. In the present study reduced GST activity in both nondiabetic atrazine treated rats and diabetic atrazine treated rats suggests that there is a reduction in reduced glutathione metabolites production. Nonsignificant elevated activities of SOD and GPx were observed in diabetic atrazine treated rats compared to diabetic control rats, which suggests that diabetic complications are not the same in each individual group or these elevated activities may be due to an increase in body weight and testicular weight in each group of animals.

The rate limiting step in steroidogenesis is translocation of cholesterol from outer membrane to inner membrane of mitochondria in testis. 3*β*-HSD and 17*β*-HSD play an important role in steroidogenesis. 3*β*-HSD converts dehydroepiandrosterone (DHEA) to androstenedione. 17*β*-HSD converts androstenedione to testosterone. The cytochrome p450 enzymes produce free radicals by formation of pseudosubstrate-O_2_ complex [45, 46] and these free radicals interact with steroid products. Increased production of H_2_O_2_ decreases activities of steroidogenic enzymes. Antiandrogenic effects of H_2_O_2_ were demonstrated in mouse Leydig tumor cells [46, 47]. Increased free radical production is linked to reduced steroidogenesis. Reports are suggesting androgenic dehydrogenases are significantly inhibited by free radicals [48–51]. Exposure of atrazine at higher doses leds to reduced expressions of 3*β*-HSD and 17*β*-HSD genes [52]. In the present study reduction in 3*β*-HSD and 17*β*-HSD activities may be due to an increased production of H_2_O_2_. Increased activities of 3*β*-HSD and 17*β*-HSD in diabetic atrazine treated rats may be due to an increase in body weight and testis weight or may be due to decreased production of H_2_O_2_ and TBARS.

Normal control rats (Figure 5(a)—group I) reveal normal spermatogonia, Sertoli cells, and Leydig cells with accumulation of spermatozoa within the seminiferous tubules. Animals treated with atrazine at a dose of 120 mg/Kg bw showing reduction in Leydig cell size, irregular shapes, and degenerative changes were observed [53]. In the present study normal atrazine treated rats (Figure 5(b)—group II) show normal spermatogonia, Sertoli cells, and Leydig cells with accumulation of spermatozoa within the seminiferous tubules because the dosage of atrazine (300 *μ*g/kg bw) is very low. Experimentally induced diabetic rats show disturbed spermatogenesis, dilated seminiferous tubules, and decreased numbers of spermatogonium, primary spermatocytes, spermatids, and mature spermatozoa [54]. In this study, diabetic control rats (Figure 5(c)—group III) show reduced numbers of spermatogonia, Sertoli cells, and Leydig cells with drastic reduction of spermatozoa within the seminiferous tubules. Diabetic atrazine treated animals (Figure 5(d)—group IV) reveal reduced numbers of spermatogonia, Sertoli cells, and Leydig cells with drastic reduction of spermatozoa within the seminiferous tubules; this group was showing histology similar to diabetic control because the dosage of atrazine (300 *μ*g/kg) is very low.

In conclusion exposure to atrazine at a dosage of 300 *μ*g/kg body weight has no significant effects on blood glucose levels, testicular antioxidant status, sperm functions, and steroidogenic enzyme activities and histology also reveals that atrazine at a dosage of 300 *μ*g/kg body weight does not acquire reproductive toxicity. To know the effects of atrazine in diabetic rats further studies have to be carried out with increased concentration of atrazine.

## Figures and Tables

**Table 1:** Effect of atrazine on body and testis weight.

Parameters	NC	NA	DC	DA
Initial body weight	163.33 ± 05.77	170.66 ± 05.13	140.00 ± 08.75	152.25 ± 05.90
Final body weight	242.33 ± 14.90	272.66 ± 21.00	193.25 ± 18.82	217.75 ± 19.60
Testis weight	1.22 ± 0.11	1.45 ± 0.15	1.2 ± 0.22	1.27 ± 0.19

The data are represented as mean ± SD (*n* = 4) and evaluated by one-way analysis of variance (ANOVA) confirmed that the groups are not significantly differed (*P* > 0.05). NC: nondiabetic control rats, NA: nondiabetic atrazine treated rats, DC: diabetic control rats, and DA: diabetic atrazine treated rats.

**Table 2:** Effect of atrazine on sperm parameters.

Parameters	NC	NA	DC	DA
Sperm count (×10^6^)	79.40 ± 06.45	72.60 ± 05.90	60.5 0 ± 04.71	59.30 ± 04.39
Sperm motility (%)	89.73 ± 06.37	84.88 ± 04.32	69.78 ± 05.98	66.33 ± 03.41
Viability (%)	96.80 ± 04.31	89.11 ± 05.03	62.66 ± 04.20	63.69 ± 04.31

The data are represented as mean ± SD (*n* = 4) and evaluated by one-way analysis of variance (ANOVA) confirmed that the groups are not significantly differed (*P* > 0.05). NC: nondiabetic control rats, NA: nondiabetic atrazine treated rats, DC: diabetic control rats, and DA: diabetic atrazine treated rats.

**Table 3:** Effect of atrazine on blood glucose levels and antioxidant status.

Parameters	NC	NA	DC	DA
Blood glucose^a^	89.73 ± 1.05	90.89 ± 3.04	255.33 ± 9.60	268.67 ± 2.89
CAT^b^	10.05 ± 1.01	8.30 ± 0.87	8.10 ± 0.75	7.90 ± 0.78
SOD^c^	0.41 ± 0.02	0.36 ± 0.01	0.27 ± 0.01	0.28 ± 0.01
GPx^d^	12.17 ± 1.11	10.40 ± 0.94	8.00 ± 0.69	8.10 ± 0.82
GSH^e^	13.56 ± 1.05	11.03 ± 1.02	8.97 ± 0.93	8.40 ± 0.85
GST^f^	0.85 ± 0.05	0.72 ± 0.03	0.43 ± 0.04	0.40 ± 0.03

The data are represented as mean ± SD (*n* = 4) and evaluated by one-way analysis of variance (ANOVA) confirmed by Tukey's test. Significant difference (*P* < 0.05) is observed only in blood glucose levels of normal control and normal atrazine treated rats compared with diabetic control and diabetic atrazine treated rats, whereas the other parameters (CAT, SOD, GPx, GSH, and GST) are not significantly differed (*P* > 0.05). NC: nondiabetic control rats, NA: nondiabetic atrazine treated rats, DC: diabetic control rats, and DA: diabetic atrazine treated rats.

^a^mg/dL.

^b^ *μ*M of H_2_O_2_ consumed/min/mg protein.

^c^mM pyrogallol oxidized min/mg protein.

^d^ *μ*mol of glutathione (GSH) utilized/min/mg protein.

^e^ *μ*g/dL.

^f^ *μ*mol of 1-chloro-2,4 dinitrobenzene (CDNB)-glutathione(GSH) conjugated/min/mg protein.
